# Operational effectiveness of tafenoquine and primaquine for the prevention of *Plasmodium vivax* recurrence in Brazil: a retrospective observational study

**DOI:** 10.1016/S1473-3099(24)00074-4

**Published:** 2024-06

**Authors:** Marcelo Brito, Rosilene Rufatto, José Diego Brito-Sousa, Felipe Murta, Vanderson Sampaio, Patrícia Balieiro, Djane Baía-Silva, Vanessa Castro, Brenda Alves, Aline Alencar, Stephan Duparc, Penny Grewal Daumerie, Isabelle Borghini-Fuhrer, Elodie Jambert, Cássio Peterka, Francisco Edilson Lima, Leonardo Carvalho Maia, Catherine Lucena Cruz, Bruna Maciele, Mariana Vasconcelos, Myrna Machado, Elder Augusto Figueira, Antônio Alcirley Balieiro, Dhelio Batista Pereira, Marcus Lacerda

**Affiliations:** a Fundação de Medicina Tropical Doutor Heitor Vieira Dourado, Manaus, Brazil; b Centro de Pesquisa em Medicina Tropical de Rondônia, Porto Velho, Brazil; c Universidade do Estado do Amazonas, Manaus, Brazil; d Medicines for Malaria Venture, Geneva, Switzerland; e Brazilian Ministry of Health, Brasília, Brazil; f Fundação de Vigilância em Saúde, Manaus, Brazil; g Instituto Leônidas & Maria Deane, Fiocruz, Manaus, Brazil; h University of Texas Medical Branch, Galveston, TX, USA

## Abstract

**Background:**

Prevention of *Plasmodium vivax* malaria recurrence is essential for malaria elimination in Brazil. We evaluated the real-world effectiveness of an updated treatment algorithm for *P vivax* radical cure in the Brazilian Amazon.

**Methods:**

In this non-interventional observational study, we used retrospective data from the implementation of a *P vivax* treatment algorithm at 43 health facilities in Manaus and Porto Velho, Brazil. The treatment algorithm consisted of chloroquine (25 mg/kg over 3 days) and point-of-care quantitative glucose-6-phosphate dehydrogenase (G6PD) testing followed by single-dose tafenoquine 300 mg (G6PD normal, aged ≥16 years, not pregnant and not breastfeeding), 7-day primaquine 0·5 mg/kg per day (G6PD intermediate or normal, aged ≥6 months, not pregnant, and not breastfeeding or breastfeeding for >1 month), or primaquine 0·75 mg/kg per week for 8 weeks (G6PD deficient, aged ≥6 months, not pregnant, and not breastfeeding or breastfeeding for >1 month). *P vivax* recurrences were identified from probabilistic linkage of routine patient records from the Brazilian malaria epidemiological surveillance system. Recurrence-free effectiveness at day 90 and day 180 was estimated using Kaplan–Meier analysis and hazard ratios (HRs) by multivariate analysis. This clinical trial is registered with ClinicalTrials.gov, NCT05096702, and is completed.

**Findings:**

Records from Sept 9, 2021, to Aug 31, 2022, included 5554 patients with *P vivax* malaria. In all treated patients of any age and any G6PD status, recurrence-free effectiveness at day 180 was 75·8% (95% CI 74·0–77·6) with tafenoquine, 73·4% (71·9–75·0) with 7-day primaquine, and 82·1% (77·7–86·8) with weekly primaquine. In patients aged at least 16 years who were G6PD normal, recurrence-free effectiveness until day 90 was 88·6% (95% CI 87·2–89·9) in those who were treated with tafenoquine (n=2134) and 83·5% (79·8–87·4) in those treated with 7-day primaquine (n=370); after adjustment for confounding factors, the HR for recurrence following tafenoquine versus 7-day primaquine was 0·65 (95% CI 0·49–0·86; p=0·0031), with similar outcomes between the two treatments at day 180 (log-rank p=0·82). Over 180 days, median time to recurrence in patients aged at least 16 years who were G6PD normal was 92 days (IQR 76–120) in those treated with tafenoquine and 68 days (52–94) in those treated with 7-day primaquine.

**Interpretation:**

In this real-world setting, single-dose tafenoquine was more effective at preventing *P vivax* recurrence in patients aged at least 16 years who were G6PD normal compared with 7-day primaquine at day 90, while overall efficacy at 180 days was similar. The public health benefits of the *P vivax* radical cure treatment algorithm incorporating G6PD quantitative testing and tafenoquine support its implementation in Brazil and potentially across South America.

**Funding:**

Brazilian Ministry of Health, Municipal and State Health Secretariats; Fiocruz; Medicines for Malaria Venture; Bill & Melinda Gates Foundation; Newcrest Mining; and the UK Government.

**Translation:**

For the Portuguese translation of the abstract see Supplementary Materials section.

## Introduction

In 2022, there were 552 000 malaria cases in the WHO region of the Americas, of which 72% were caused by *Plasmodium vivax*.^1^ The *P vivax* parasite lifecycle includes a dormant hypnozoite which can reactivate to cause multiple clinical relapses and promote onward transmission. Complete treatment (radical cure) requires a blood schizonticide drug to clear acute blood-stage infection and an 8-aminoquinoline, either primaquine or tafenoquine, to clear hypnozoites from the liver and prevent relapse.

In Brazil, a 7-day (0·5 mg/kg per day) primaquine regimen has been used since the mid-1990s to promote adherence.^2^ However, unsupervised adherence to 7-day primaquine is as low as 67% in some populations.^2^, ^3^ In October, 2019, the Brazilian Health Regulatory Agency registered tafenoquine, a single-dose 8-aminoquinoline. In clinical trials, tafenoquine plus chloroquine reduced *P vivax* recurrence by 70% compared with chloroquine plus placebo,^4^ with similar recurrence-free efficacy to chloroquine plus 14-day primaquine.^4^, ^5^ Both primaquine and tafenoquine can cause haemolysis in individuals with glucose-6-phosphate dehydrogenase (G6PD) enzyme deficiency, which might lead to acute haemolytic anaemia; therefore, G6PD testing is required before administration of these drugs.^6^, ^7^

**Research in context**

**Evidence before this study**

We searched PubMed on Sept 25, 2023, for publications in any language, using the terms [tafenoquine] AND [vivax] AND [relapse], and found 89 results. Six studies were clinical trials, two of which were phase 3, double-blind, double-dummy, randomised placebo-controlled trials in patients aged at least 16 years with *Plasmodium vivax* malaria and normal glucose-6-phosphate dehydrogenase (G6PD) enzyme activity who received chloroquine plus placebo, chloroquine plus single-dose tafenoquine, or chloroquine plus 14-day primaquine. One trial showed a significantly lower risk of recurrence after 6 months with chloroquine plus tafenoquine compared with chloroquine plus placebo (hazard ratio 0·30, 95% CI 0·22–0·40; p<0·001). In the other trial, the proportion of patients who were free from recurrence at 6 months was 67·0% (95% CI 61·0–72·3) in the 426 patients in the tafenoquine group and 72·8% (65·6–78·8) in the 214 patients in the 14-day primaquine group. There was no difference in haematological endpoints between 14-day primaquine and tafenoquine in these patients who were G6PD normal. The relative effect of adherence to single-dose tafenoquine versus 14-day primaquine on clinical efficacy was not assessed. No operational studies of tafenoquine were identified and single-dose tafenoquine has not been assessed in a real-world setting.

**Added value of this study**

This exploratory analysis examined *P vivax* recurrences after treatment with single-dose tafenoquine or 7-day primaquine following implementation of a new treatment algorithm for *P vivax* radical cure in Brazil. The comprehensive capture of all malaria cases within Brazil in the malaria surveillance system and the capacity to link malaria patient records enabled identification of recurrences up to day 180. The study aimed to provide evidence to inform programmatically relevant endpoints for patient follow-up and drug efficacy surveillance until day 90. However, extending the study to day 180 allowed verification of radical cure effectiveness versus efficacy data from comparative randomised clinical trials.

**Implications of all the available evidence**

The operational effectiveness of *P vivax* radical cure incorporating quantitative G6PD testing and tafenoquine in the Brazilian Amazon was consistent with efficacy data from South American sites included in randomised controlled trials of tafenoquine. The study supports the national adoption of this treatment algorithm for *P vivax* malaria radical cure in Brazil and potentially across South America.

Brazil is targeting malaria elimination by 2035 and effective *P vivax* radical cure is needed to achieve this goal. In 2021, the Brazilian Ministry of Health developed a revised treatment algorithm for *P vivax* radical cure, incorporating quantitative G6PD testing and single-dose tafenoquine. This algorithm was initially implemented within the Tafenoquine Rollout STudy (TRuST) conducted across two municipalities of the Brazilian Amazon. TRuST showed that quantitative G6PD screening and tafenoquine treatment were highly feasible within the Brazilian health system with acceptable haemolytic risk.^8^ Thus, there is the potential to provide *P vivax* radical cure to adults who are G6PD normal with a single-dose treatment plus standard 3-day chloroquine.

To understand the programmatic impact of the new treatment algorithm, it was important to assess treatment effectiveness for preventing *P vivax* recurrence to inform appropriate patient follow-up. In Thailand, follow-up at 90 days has been challenging to implement, and might not be appropriate in Brazil.^9^ Follow-up at 60 days would increase feasibility. In this exploratory analysis, we used retrospective data from the Brazilian malaria epidemiological surveillance system (SIVEP-Malaria) for the centres included in TRuST to estimate recurrence-free effectiveness over 90 days. Additionally, we extended the analysis to 180 days to compare operational effectiveness with clinical efficacy data from the South American centres included in two published phase 3 randomised clinical studies.^4^, ^5^

## Methods

### Study design

Full details of the TRuST study and the protocol have been published.^8^ TRuST was a non-interventional, observational study based on the analysis of secondary data routinely collected and recorded in SIVEP-Malaria. In Brazil, as a matter of policy, malaria can only be treated within the public health system, and SIVEP-Malaria includes data for all patients who have had a diagnostic test for malaria, including their results, treatment, and outcomes, representing a comprehensive resource for operational research.^8^ The treatment algorithm was implemented at 43 health-care centres between September, 2021, and August, 2022, in Manaus (Amazonas State) and Porto Velho (Rondônia State). Manaus has a population of around 2·1 million people, with an annual malaria incidence per 1000 population at risk of 2·18 in 2021 and 1·38 in 2022. Porto Velho has a population of 460 000, with an annual malaria incidence per 1000 population at risk of 14·2 in 2021 and 15·8 in 2022.^10^

Quantitative G6PD testing and tafenoquine treatment were implemented in malaria diagnostic units within the Brazilian public health system. Primaquine and tafenoquine were supplied via the normal channels within the health system. Malaria diagnosis, G6PD screening and quality control, treatment allocation, and case notification were done by each municipality team during the implementation period.

The protocol was approved by the Brazilian National Ethics in Research Committee (CAAE 16867319.6.0000.0008). The study complied with the Declaration of Helsinki (2013) and local regulations. All participants provided informed consent to use their de-identified data. Study staff had access to SIVEP-Malaria with due authorisation with data de-identified before analysis.

### Participants

This analysis included all patients with confirmed *P vivax* malaria monoinfection or *P vivax* and *Plasmodium falciparum* mixed infection presenting between Sept 9, 2021, and Aug 31, 2022, to clinics in the implementation areas who received tafenoquine, 7-day primaquine, or weekly primaquine and provided informed consent.

### Procedures

*P vivax* was diagnosed predominantly using microscopy as per nationally defined protocols, although rapid diagnostic tests were acceptable.^11^ Demographic, laboratory, and epidemiological data were entered into SIVEP-Malaria by municipal staff as per routine surveillance protocols. Following a positive *P vivax* malaria diagnosis, a point-of-care quantitative G6PD test (STANDARD G6PD test, SD Biosensor, Suwon-si, South Korea) was performed as per manufacturer's instructions. G6PD activity was reported as a ratio to haemoglobin (U/g Hb) and defined in both males and females as deficient (≤4·0 U/g Hb), intermediate (4·1–6·0 U/g Hb), or normal (≥6·1 U/g Hb).

Drugs were chloroquine 150 mg tablets (Farmanguinhos, Fiocruz, Rio de Janeiro, Brazil), tafenoquine 150 mg tablets (GlaxoSmithKline, Ware, UK), and primaquine 5 mg and 15 mg tablets (Farmanguinhos, Fiocruz). Treatments were administered orally with the first dose supervised; further adherence data are not available. The treatment algorithm specified 3-day chloroquine for all patients (25 mg/kg over 3 days) plus 8-aminoquinolines as follows: single-dose tafenoquine (300 mg) for patients aged at least 16 years who were G6PD normal (non-pregnant and non-breastfeeding); 7-day primaquine (0·5 mg/kg per day) for patients aged at least 6 months who were G6PD normal or intermediate (non-pregnant and non-breastfeeding or breastfeeding for >1 month); and weekly primaquine (0·75 mg/kg per week) for 8 weeks for patients aged at least 6 months who were G6PD deficient (non-pregnant and non-breastfeeding or breastfeeding for >1 month). Pregnant women, women breastfeeding for 1 month or less, and children younger than 6 months were excluded from 8-aminoquinoline therapy.

### Data acquisition

*P vivax* recurrences were identified as previously described.^12^ Data for eligible patients were extracted from the SIVEP-Malaria forms. As each malaria episode is treated as a new case, *P vivax* recurrences in the same individual were identified by applying a probabilistic function using patient's name, birth date, mother's name, and malaria notification unit, and automatically verified, applying a probability threshold (>0·7), followed by further individual linkage of discrepancies. Linkages were sought for 180 days after the end of treatment for the initial malaria episode. Because recurrences occurring within 5 days of treatment initiation were considered worsening caused by severe malaria, at least a 5-day period between the first diagnosis and a new positive smear was considered a recurrence.

### Outcomes

The primary objective of this exploratory analysis was to generate evidence to support operationally relevant endpoints that would allow assessment of patient outcomes and drug effectiveness surveillance. Although patient outcomes can be assessed up to a maximum of 90 days, 60-day follow-up would increase feasibility. A comparative analysis was done between single-dose tafenoquine and 7-day primaquine to assess recurrence-free effectiveness until day 90 overall and separately for Manaus and Porto Velho. Because the treatment algorithm specified different patient populations for tafenoquine and 7-day primaquine, the analysis population was defined as patients aged at least 16 years who were G6PD normal. This analysis relied on a proportion of patients being treated with 7-day primaquine contrary to the treatment algorithm.

Two phase 3 studies of tafenoquine involved centres in South America, including Brazil, with a primary outcome of recurrence-free efficacy at 6 months in adult patients who were G6PD normal.^4^, ^5^ To verify operational effectiveness, we extended our analysis to evaluate recurrence-free effectiveness of single-dose tafenoquine and 7-day primaquine in patients aged at least 16 years who were G6PD normal until day 180. Additionally, to provide a benchmark for the overall effectiveness of the *P vivax* radical cure treatment algorithm, an analysis was done for all treated patients (any age, any G6PD status), assessed at day 90 and day 180.

### Statistical analysis

Because this study included all consenting patients with confirmed *P vivax* malaria within Manaus and Porto Velho who received 8-aminoquinolines during the evaluation period, there was no sampling protocol and no prespecified sample size. Recurrence-free probability was estimated using Kaplan–Meier survival analysis with 95% CIs calculated using the Greenwood method (R survival library). Origin and start time were the day of presentation for the initial *P vivax* episode and end time was 180 days after treatment completion. Only the first recurrence was included, and any subsequent recurrences were excluded. There were no other criteria for censoring because all patients were followed up for the entire study period via SIVEP-Malaria reporting. The log-rank test was used (p value <0·05 significant) to evaluate the overall difference in the Kaplan–Meier curves. Median time to first recurrence with IQR was also calculated.

Visual and correlation tests (Martingale and Schoenfeld residuals) did not confirm proportional hazards for age and group, although all criteria met the assumption of linearity. Hazard ratios (HRs) were obtained using a Weibull regression model for survival data for all treated patients. The best fit was possible only up to day 90, and it was not possible to find a parametric model that fit these data until day 180. Model fit was assessed using goodness-of-fit plots, and the Wald test was used to examine the relative importance of treatment group, age, weight, race, sex, and county. On the basis of backward elimination and Akaike criteria, treatment group and age were retained to calculate the HRs for each covariate. Linearity was confirmed for age using the Ramsey regression equation specification error test (p=0·456). The model was applied to the analysis population (patients aged at least 16 years who were G6PD normal) using the same variables. However, the variable selection process found that the best model contained only treatment group. Analyses were done with R (version 4.1.3) in the integrated development environment Rstudio (version 2023.03.0). This study is registered with ClinicalTrials.gov, NCT05096702.

### Role of the funding source

The Brazilian National Malaria Programme, Fiocruz, and Medicines for Malaria Venture were involved in the protocol design, data analysis, reporting, and the decision to submit for publication. Other funders were not involved in the study design, conduct, or analysis.

## Results

**Figure 1:**
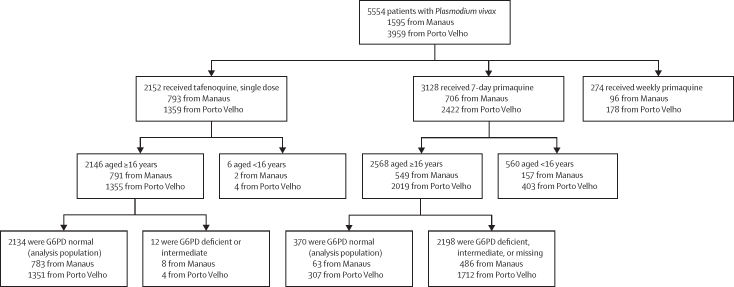
Study profile G6PD activity was reported as a ratio to haemoglobin (U/g Hb) and defined in both males and females as normal (≥6·1 U/g Hb), intermediate (4·1–6·0 U/g Hb), or deficient (≤4·0 U/g Hb). G6PD=glucose-6-phosphate dehydrogenase.

Between Sept 9, 2021, and Aug 31, 2022, 5554 participants were diagnosed with *P vivax* and received single-dose tafenoquine, 7-day primaquine, or weekly primaquine (figure 1). The analysis population comprised 2504 patients aged at least 16 years who were G6PD normal who received tafenoquine (2134 [85·2%] patients) or 7-day primaquine (370 [14·8%] patients; figure 1). Baseline data for the analysis population are shown in table 1 and for all treated patients and by municipality in appendix 2 (pp 2–4). Patient characteristics varied between the treatment groups, as would be expected from this non-selected patient population (table 1, appendix 2 pp 2–4).

**Table 1:** Socioeconomic and demographic baseline characteristics (analysis population)

		**Tafenoquine, single dose (n=2134)**	**7-day primaquine (n=370)**	p value*
Age (years)		39·5 (14·1); 39·0 (28·0–50·0)	38·3 (15·5); 36·0 (27·0–48·0)	0·029
	≥16 years	2134 (100%)	370 (100%)	..
G6PD classification				
	Deficient	0	0	..
	Intermediate	0	0	..
	Normal	2134 (100%)	370 (100%)	NA
G6PD activity (U/g Hb)		7·6 (1·4); 7·2 (6·6–8·1)	7·5 (1·5); 7·2 (6·6–8·1)	0·38
Sex				
	Female	738 (34·6%)	161 (43·5%)	..
	Male	1396 (65·4%)	209 (56·5%)	0·0009
Weight (kg)		75·4 (26·3); 72·0 (63·0–85·0)	70·7 (16·7); 70·0 (60·0–80·0)	0·0002
Race				
	Mixed	1828 (85·7%)	322 (87·0%)	..
	White	194 (9·1%)	33 (8·9%)	..
	Black	72 (3·4%)	10 (2·7%)	..
	Indigenous	24 (1·1%)	4 (1·1%)	..
	Asian	16 (0·7%)	1 (0·3%)	0·90
Municipality				
	Porto Velho	1351 (63·3%)	307 (83·0%)	..
	Manaus	783 (36·7%)	63 (17·0%)	<0·0001
Facility level				
	High complexity	437 (20·5%)	20 (5·4%)	..
	Medium complexity	1377 (64·5%)	292 (78·9%)	..
	Low complexity	320 (15·0%)	58 (15·7%)	<0·0001
Education†				
	Missing‡	14	12	..
	Illiterate	36 (1·7%)	5 (1·4%)	..
	1st to 5th incomplete	279 (13·2%)	37 (10·3%)	..
	4th elementary school completed	80 (3·8%)	15 (4·2%)	..
	5th to 9th elementary school incomplete	313 (14·8%)	72 (20·1%)	..
	Elementary school completed	325 (15·3%)	28 (7·8%)	..
	Incomplete high school	350 (16·5%)	64 (17·9%)	..
	Completed high school	583 (27·5%)	111 (31·0%)	..
	Incomplete college	46 (2·2%)	6 (1·7%)	..
	Completed college education	108 (5·1%)	20 (5·6%)	0·0046
Occupation in past 15 days				
	Missing‡	79	10	..
	Agriculture	135 (6·6%)	34 (9·4%)	..
	Cattle	3 (0·1%)	1 (0·3%)	..
	At home	348 (16·9%)	115 (31·9%)	..
	Tourism	90 (4·4%)	40 (11·1%)	..
	Gold mining	32 (1·6%)	3 (0·8%)	..
	Plant exploration	33 (1·6%)	8 (2·2%)	..
	Hunting or fishing	14 (0·7%)	6 (1·7%)	..
	Construction roads or dams	3 (0·1%)	1 (0·3%)	..
	Mining	4 (0·2%)	2 (0·6%)	..
	Traveller	37 (1·8%)	9 (2·5%)	..
	Other	1356 (66·0%)	141 (39·2%)	<0·0001

Data are n (%) or mean (SD); median (IQR). G6PD=glucose-6-phosphate dehydrogenase. NA=not applicable.

* Kruskal–Wallis rank sum test; Pearson's χ^2^ test.

† Educational years in Brazil are as follows: elementary is primary years 1 to 5 (age 6–10 years) and lower secondary years 6 to 9 (age 11–14 years); high school is upper secondary (age 15–17 years); college is post-secondary education (age ≥18 years).

‡ Missing values were not included in the denominator for percentage calculations.

Kaplan–Meier curves must be interpreted with caution because for *P vivax* malaria, recrudescence (chloroquine failure), relapses (8-aminoquinoline failure), and reinfections cannot be distinguished clinically or by molecular methods.^13^ However, the analysis provides insights into relevant programmatic outcomes.

In the analysis population, two (0·1%) of 2134 patients prescribed tafenoquine and one (0·3%) of 370 prescribed 7-day primaquine had recurrence by day 30. In Brazil, recurrences before day 28 are considered chloroquine failures and there was no clinical evidence of chloroquine resistance.

**Figure 2:**
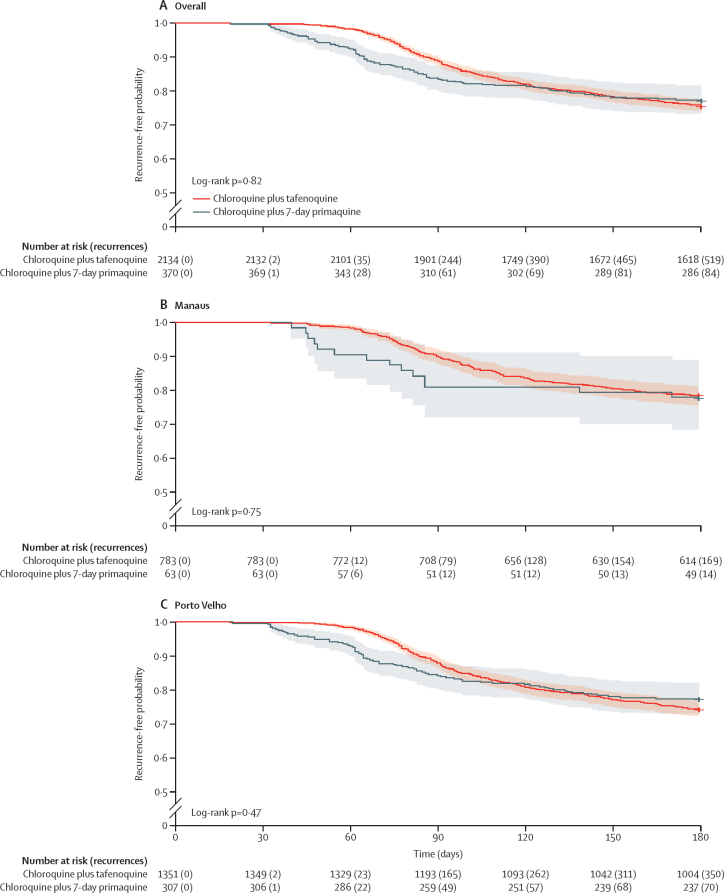
*Plasmodium vivax* recurrence following chloroquine plus single-dose tafenoquine or 7-day primaquine (analysis population) The shaded area for each line represents the 95% CI.

Between days 30 and 60, the two Kaplan–Meier curves separated (log-rank p=0·37 at day 30 and p<0·0001 at day 60; figure 2A). This difference is operationally relevant because recurrences before day 60 are treated with 14-day primaquine (0·5 mg/kg per day) plus an artemisinin-based combination therapy, whereas recurrences after day 60 are treated with 7-day primaquine or single-dose tafenoquine. A decline in day 60 effectiveness could be a reasonable metric for evaluating drug resistance because it would be most sensitive to changes in relapse prevention effectiveness (rather than reinfection) and is feasible for routine surveillance.

The difference between the tafenoquine and 7-day primaquine curves was maintained until at least day 90 (log-rank p=0·0024; figure 2A), with similar findings observed for Manaus (log-rank p=0·019; figure 2B) and Porto Velho (log-rank p=0·042; figure 2C). Until day 90, estimated recurrence-free effectiveness of tafenoquine was 88·6% (95% CI 87·2–89·9) compared with 83·5% (79·8–87·4) for 7-day primaquine (table 2). At day 90, median time to recurrence was 75 days (IQR 66–82) in patients treated with tafenoquine and 62 days (46–74) in patients treated with 7-day primaquine. After adjustment for covariates, the overall HR for recurrence following tafenoquine versus 7-day primaquine was 0·65 (95% CI 0·49–0·86; p=0·0031) at day 90.

**Table 2:** Estimated recurrence-free effectiveness of single-dose tafenoquine and 7-day primaquine until day 90 and day 180

		**Day 90**		**Day 180**	
		Tafenoquine, single dose	7-day primaquine	Tafenoquine, single dose	7-day primaquine
Analysis population (age ≥16 years, G6PD normal)		88·6% (87·2–89·9)	83·5% (79·8–87·4)	75·7% (73·9–77·5)	77·3% (73·1–81·7)
	Manaus	89·9% (87·8–92·0)	81·0% (71·8–91·3)	78·4% (75·6–81·4)	77·8% (68·2–88·8)
	Porto Velho	87·8% (86·1–89·6)	84·0% (80·0–88·2)	74·1% (71·8–76·5)	77·2% (72·6–82·0)
All treated patients (any age, any G6PD status)		88·6% (87·3–90·0)	81·8% (80·5–83·2)	75·8% (74·0–77·6)	73·4% (71·9–75·0)
	Manaus	89·9% (87·8–92·0)	83·1% (80·4–86·0)	78·6% (75·8–81·5)	77·2% (74·2–80·4)
	Porto Velho	87·9% (86·1–89·6)	81·5% (79·9–83·0)	74·2% (71·9–76·5)	72·3% (70·6–74·1)

Values are % probability derived from Kaplan–Meier analysis (95% CI). G6PD=glucose-6-phosphate dehydrogenase.

The two Kaplan–Meier lines converged around day 120 (log-rank p=0·56) and were equivalent until day 180 (log-rank p=0·82; figure 2A). The pattern was similar for Manaus (log-rank p=0·75; figure 2B) and Porto Velho (log-rank p=0·47; figure 2C), although the two lines converged slightly later in Manaus than in Porto Velho, possibly reflecting the higher malaria transmission rate in Porto Velho. Until day 180, estimated recurrence-free effectiveness was 75·7% (95% CI 73·9–77·5) with tafenoquine and 77·3% (73·1–81·7) with 7-day primaquine. Over 180 days, median time to recurrence was 92 days (IQR 76–120) with tafenoquine and 68 days (52–94) with 7-day primaquine. Findings for Manaus and Porto Velho were consistent (appendix 2 p 5). These results suggest that to ensure patient outcomes, follow-up until at least day 90 is needed. This would capture most recurrences caused by relapse across both aminoquinolines.

**Figure 3:**
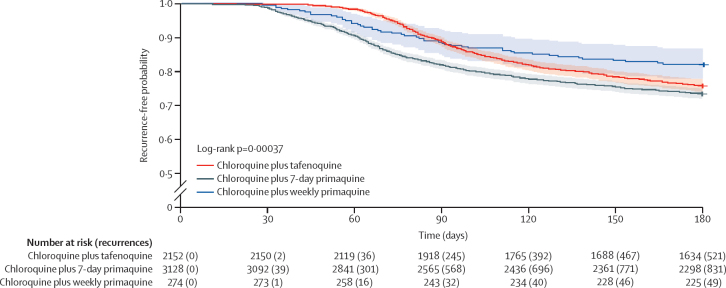
*Plasmodium vivax* recurrence following chloroquine plus single-dose tafenoquine, 7-day primaquine, or weekly primaquine (all treated patients) The shaded area for each line represents the 95% CI.

An analysis for all treated patients compared the overall effectiveness of the treatment algorithm (figure 3). Across all patients of any age and any G6PD status, there was a difference between the tafenoquine and 7-day primaquine curves until day 180 (log-rank p=0·0044) and between tafenoquine, 7-day primaquine, and weekly primaquine until day 180 (log-rank p=0·00037). Although the patient populations for the three treatments were not comparable, we can conclude that until day 180, the overall effectiveness of the treatment algorithm exceeded 73%, and tafenoquine was at least as effective as the standard 7-day primaquine regimen used in Brazil. This analysis also showed the effectiveness of treating patients with *P vivax* who were G6PD deficient with weekly primaquine. At day 180, estimated recurrence-free effectiveness was 75·8% (95% CI 74·0–77·6) with tafenoquine, 73·4% (71·9–75·0) with 7-day primaquine, and 82·1% (77·7–86·8) with weekly primaquine.

Across all treated patients, recurrence-free effectiveness at day 90 was 88·6% (95% CI 87·3–90·0) with tafenoquine, 81·8% (80·5–83·2) with 7-day primaquine, and 88·3% (84·6–92·2) with weekly primaquine. Multivariate analysis comparing recurrence-free effectiveness until day 90 with tafenoquine and 7-day primaquine, adjusting for treatment group, age, weight, race, sex, and municipality, indicated a clinically relevant effect of treatment group on recurrence, with an HR of 0·60 (95% CI 0·51–0·69; p<0·0001) and a statistically significant, but not clinically relevant, effect of age: HR 0·995 (0·991–0·999; p=0·032).

## Discussion

In this real-life investigation of *P vivax* radical cure in the Brazilian Amazon, in patients aged at least 16 years who were G6PD normal, single-dose tafenoquine was significantly more effective at preventing *P vivax* recurrence than 7-day primaquine over 90 days following treatment initiation. However, by day 180, the probability of recurrence was similar for the two treatments.

Recurrence was delayed with tafenoquine to around 92 days versus 68 days with primaquine. This delay in recurrence is clinically relevant because patients with *P vivax* malaria have a large parasite biomass in the erythropoietic organs, provoking anaemia which deepens with each successive episode.^14^ Thus, a longer period between episodes allows patients more time to recover. In a placebo-controlled trial, patients treated with tafenoquine or 14-day primaquine had haematological recovery by around day 60, whereas those treated with chloroquine alone did not recover until day 120.^4^ Also, the delay in recurrence with tafenoquine has programmatic implications regarding the treatment frequency and the type of treatment, because recurrences before day 60 are treated with 14-day primaquine (0·5 mg/kg per day) plus artemisinin-based combination therapy. The data from this study will inform cost-effectiveness modelling to investigate the potential impacts of these differences at day 90.

There are several potential explanations for the delay in *P vivax* recurrence observed for tafenoquine versus 7-day primaquine. Treatment adherence has a major role in effectiveness of *P vivax* radical cure in the Amazon,^2^, ^3^ and data from Brazil show that *P vivax* recurrence is delayed with supervised 7-day primaquine compared with unsupervised 7-day primaquine.^15^ These findings suggest that the delay in recurrence observed with tafenoquine could result, at least in part, from improved adherence to the single-dose regimen. Another consideration is that individuals with cytochrome P450 2D6 (CYP2D6) polymorphisms conferring reduced enzyme activity have an increased recurrence risk following primaquine, as observed in individuals from the Brazilian Amazon.^16^ By contrast, clinical evidence for tafenoquine suggests that CYP2D6 activity does not influence efficacy.^17^ Pharmacokinetics are also a consideration. The tafenoquine half-life is about 15–16 days,^18^ whereas primaquine has a half-life of 6 h, requiring multiple dosing. However, because both drugs have unknown metabolites active against hypnozoites, further work is needed to determine the mechanism of action, pharmacokinetics, and pharmacodynamics of 8-aminoquinolines and the potential effect on post-treatment prophylaxis and the suppression of early recurrent parasitaemia.^17^

We considered recurrences to day 180 in patients aged at least 16 years with *P vivax* malaria who were G6PD normal to allow comparison with the two tafenoquine phase 3 clinical trials that used a 6-month endpoint and a similar population. In these two clinical trials, recurrence-free efficacy of tafenoquine in the South American centres was 68·4% (95% CI 58·3–76·6) and 63·3% (53·8–71·4) at 6 months.^4^, ^5^ In our study, recurrence-free effectiveness with tafenoquine was higher at day 180 (75·7%, 95% CI 73·9–77·5). Similarly, we found that recurrence-free effectiveness at day 180 for 7-day primaquine (77·3%, 95% CI 73·1–81·7) was higher than recurrence-free efficacy of 14-day primaquine (15 mg per day) at 6 months in the phase 3 clinical trials (68·6% [95% CI 54·9–79·0] and 60·5% [48·0–71·0]).^4^, ^5^ Thus, both single-dose tafenoquine and 7-day primaquine performed at least as well operationally as observed in the phase 3 randomised clinical trials.

Around 24% of patients had recurrence by day 180 with tafenoquine or 7-day primaquine. Ideally, *P vivax* radical cure should eliminate hypnozoites and prevent all relapses, with recurrences caused by reinfection only. In view of the transmission setting in Brazil, it is likely that a proportion of the recurrences were caused by relapse. These 8-aminoquinoline failures could be due to differences in parasite susceptibility, host immunity, or the density of hypnozoites in an individual.^19^ The timing of treatment relative to the parasite lifecycle has recently been shown to affect the persistence of hypnozoites following treatment with primaquine, with established hypnozoites surviving but with delayed reactivation.^20^ Also, low drug exposures, caused by poor adherence, poor metabolisation, or impaired bioavailability could increase relapse risk. These factors could potentially be overcome, at least in part, by increasing drug doses. For example, a study in Brazil comparing fully supervised primaquine 0·5 mg/kg per day for 7 days versus 14 days reported an increase in recurrence-free efficacy over 168 days from 59% (95% CI 47–69) for the 7-day regimen to 86% (76–92) for the 14-day regimen.^21^ However, haemolytic risk with primaquine doses more than 0·5 mg/kg per day might require restriction to patients who are G6PD normal.^22^ For tafenoquine, a dose–response model suggested that increasing the dose to 450 mg would result in a recurrence rate of around 6·2% at 4 months, compared with 15·3% (95% CI 9·6–21·7) with the 300 mg dose.^17^ However, 8-aminoquinoline dose optimisation will depend on the transmission setting and the characteristics of the endemic *P vivax* strain, with South American strains being genetically distinct from parasite populations in other endemic regions, including those in southeast Asia.^23^ The haematological risk might also vary depending on the predominant G6PD variants. Thus, controlled randomised studies assessing the safety and efficacy of different tafenoquine doses need to be done in specific malaria-endemic regions.

Nevertheless, *P vivax* radical cure is a key public health intervention. In Brazil, recurrence-free efficacy with chloroquine alone over 6 months has been reported as 25·8% (95% CI 16·1–36·7).^4^ Consequently, in this real-world study, across the treatment algorithm, *P vivax* radical cure reduced recurrences by nearly three times—a substantial reduction in *P vivax* malaria burden. Note that because only the first recurrence was considered in this study, the effect of the 8-aminoquinolines on the prevention of repeated recurrences is underestimated. Also, because *P vivax* gametocytes are produced before clinical symptoms manifest, recurrence prevention is a key intervention for malaria elimination to interrupt transmission. Because tafenoquine, and to a lesser extent primaquine, have contributory schizonticidal activity, radical cure is also incidental combination therapy for acute *P vivax* malaria.^17^, ^24^, ^25^

The comparison of tafenoquine and 7-day primaquine in patients aged at least 16 years who were G6PD normal was only possible because some patients who should have received tafenoquine were administered 7-day primaquine, contrary to the treatment algorithm. This situation arose because of an initial reluctance of some clinicians to adopt tafenoquine. This analysis provides evidence of tafenoquine effectiveness and will be a valuable tool in supporting behaviour change during national implementation of the treatment algorithm. Additionally, the study provides reassurance of the effectiveness of weekly primaquine for *P vivax* radical cure in patients who are G6PD deficient, which should encourage health workers and patients to prescribe and adhere to this protocol.

This exploratory investigation of the real-world implementation of *P vivax* radical cure has limitations relating to the retrospective design and the unselected population. Confounders were considered in the multivariate analysis, but it is possible that other factors might influence the outcome, such as vector control measures or occupational-based risks for reinfection.^26^ No information was available on CYP2D6 polymorphisms or adherence.

Tafenoquine implementation in Brazil is essential for malaria elimination.^27^ The TRuST study concluded that quantitative G6PD testing followed by *P vivax* radical cure was highly feasible in the Brazilian Amazon with no concerning risk for acute haemolytic anaemia.^8^ The current study showed the effectiveness of tafenoquine relative to 7-day primaquine and the overall effectiveness of the treatment algorithm, supporting the widespread adoption of quantitative G6PD testing and tafenoquine for *P vivax* radical cure across malaria-endemic regions in Brazil and potentially across South America.

## Data sharing

De-identified participant data are available on reasonable request and with completion of a signed data access agreement form (https://www.mmv.org/about-us/contact-us), referencing this publication. Data will be available for at least 5 years from publication of this study.

## Declaration of interests

SD, PGD, IB-F, and EJ are employees of Medicines for Malaria Venture. DBP declares grants from Butantan, PATH, and Janssen, payment for lectures or presentations from GSK, and support for attending meetings and travel from Janssen. All other authors declare no competing interests.

## Supplementary Materials

Portuguese translation of the abstract
