# Progress towards safer and more effective radical cure of *Plasmodium vivax*

**DOI:** 10.1016/S1473-3099(24)00074-4

**Published:** 2024-03-04

**Authors:** Ric N Price

**Affiliations:** Global Health Division, Menzies School of Health Research and Charles Darwin University, Darwin, NT 0811, Australia; Mahidol-Oxford Tropical Medicine Research Unit, Faculty of Tropical Medicine, Mahidol University, Bangkok, Thailand; Centre for Tropical Medicine, Nuffield Department of Clinical Medicine, University of Oxford, Oxford, UK

*Plasmodium vivax* causes a substantial burden of disease in the Americas, the Horn of Africa, and Asia-Pacific. The parasite forms dormant liver stages (hypnozoites) that reactivate weeks to months after an initial infection causing recurrent episodes of malaria (relapses). Multiple recurrences are associated with a cumulative risk of anaemia, significant morbidity, and mortality. An estimated 60–90% of vivax malaria cases are attributable to relapses, and these drive the ongoing transmission of the parasite. The elimination of both blood and liver stage parasite (known as radical cure) is crucial for the timely elimination of *P vivax*.

In 2018, tafenoquine became the first hypnozonticidal drug to be registered in 70 years. A single dose of this slowly eliminated drug has similar efficacy in reducing *P vivax* recurrences compared with daily administration of primaquine for 7–14 days (total dose 3·5 mg/kg).^1^ However, both drugs can cause severe haemolysis in individuals with glucose-6-phosphate dehydrogenase (G6PD) deficiency. A novel semi-quantitative point-of-care G6PD test (STANDARD G6PD test, SD Biosensor, Suwon-si, South Korea) has been developed to screen vulnerable patients. Although the STANDARD G6PD test demonstrates high accuracy in controlled settings,^2^ it adds a level of complexity to diagnosis that requires adapting the way in which antimalarial treatment is delivered.

Marcelo Brito and colleagues^3^ conducted a large observational study in Brazil to determine the operational feasibility of using the STANDARD G6PD test for patients with *P vivax* malaria. The trial enrolled 6075 patients with confirmed vivax malaria across 43 health facilities in Manaus and Porto Velho. Patients were allocated to either a single dose of tafenoquine, daily primaquine for 7 days, or weekly primaquine for 8 weeks, depending on the result of their G6PD test. The results highlighted excellent adherence to protocols in both high-level and low-level health-care facilities. In *The Lancet Infectious Diseases*, the investigators provide an additional retrospective analysis of data from the same study to determine the effectiveness of tafenoquine and unsupervised primaquine in preventing recurrent *P vivax* malaria.^4^

Recurrent infections were detected 24 days earlier in patients treated with 7-day primaquine compared with those treated with tafenoquine, and at 3 months there was a modest reduction in recurrence-free effectiveness (83·5% in the 7-day primaquine group *vs* 88·6% in the tafenoquine group). Treatment was not randomly assigned, but the difference remained significant after controlling for confounding baseline factors. By 6 months, the survival curves had converged, with an overall risk of recurrence of 24–27%, almost three times lower than that reported previously in patients at the same location who did not receive radical cure.^1^ Thus, the revised radical cure treatment strategy proved feasible and safe, with potential for substantial public health benefit. The investigators offer several plausible reasons for the early differences in recurrences, the most likely of which is the long post-treatment prophylaxis suppressing parasite growth after tafenoquine (half-life about 12 days) compared with primaquine (half-life about 6 h). By day 60, when blood concentrations had fallen below the minimum inhibitory concentration, the risks of recurrence began to converge. Hence, tafenoquine might delay rather than reduce the total number of recurrences. The distinction is important since it determines the impact on transmission. The investigators suggest that even a delay in recurrence is clinically relevant because it allows additional time for haematological recovery. A similar effect has been observed following schizontocidal treatment with slowly eliminated drugs versus rapidly eliminated drugs.^5^

True anti-relapse effectiveness is difficult to determine because it is not possible to distinguish whether recurrences are due to reinfection, recrudescence, or relapse.^6^ Effectiveness is confounded further by the biasing of provider and patient adherence within the context of a formal clinical trial. The study by Brito and colleagues addressed this by aiming for a real-life scenario, with minimal trial interventions including active follow-up; recurrent *P vivax* infections were detected when patients presented to the public health service using routine data collected by an excellent malaria surveillance system (SIVEP-Malaria). However, this approach will have missed recurrences with mild or no clinical symptoms; hence, the effectiveness against recurrent parasitaemia could be substantially lower than that presented for clinical disease.

A recent meta-analysis highlights that a high total dose of primaquine (7 mg/kg) is needed to achieve radical cure reliably.^7^ Tafenoquine was benchmarked against a low total dose of primaquine (3·5 mg/kg) and a complementary meta-analysis suggests that a modest increase in the dose of tafenoquine (to 7·5 mg/kg) could reduce the risk of *P vivax* recurrences by 90%.^8^ Prospective clinical trials are underway to investigate the safety and efficacy of higher doses of tafenoquine.^9,10^

Brito and colleagues should be congratulated in leading the way to explore the operational feasibility of novel tools and strategies for the radical cure of *P vivax*. In the coming years, the malaria community is well placed to optimise the delivery of these tools even further, bringing the prospect of *P vivax* elimination ever closer.
