# Supplementary material for: Operational effectiveness of tafenoquine and primaquine for the prevention of Plasmodium vivax recurrence in Brazil: a retrospective observational study
Source: Lancet Infect Dis. 2024 Jun;24(6):629–38. doi: 10.1016/S1473-3099(24)00074-4 (PMC7615970; doi:10.1016/S1473-3099(24)00074-4)
Supplement: Supplementary file 1 [file mmc1.pdf]

THE LANCET
Infectious Diseases

Supplementary appendix 1

This translation in Portuguese was submitted by the authors and we reproduce it as supplied. It has not been peer reviewed. *The Lancet*'s editorial processes have only been applied to the original in English, which should serve as reference for this manuscript.

Esta tradução em português foi submetida pelos autores e nós não fizemos quaisquer alterações. Esta versão não foi revista por pares. O processo editorial do The Lancet só foi aplicado à versão original em inglês, que deve servir como referência para este artigo.

Supplement to: Brito M, Rufatto R, Brito-Sousa JD, et al. Operational effectiveness of tafenoquine and primaquine for the prevention of *Plasmodium vivax* recurrence in Brazil: a retrospective observational study. *Lancet Infect Dis* 2024; published online March 4. https://doi.org/10.1016/S1473-3099(24)00074-4.

RESUMO

Background A prevenção da recorrência da malária por *Plasmodium vivax* é essencial para a eliminação da malária no Brasil. Avaliamos a eficácia no mundo real de um algoritmo de tratamento atualizado para a cura radical do *P vivax* na Amazônia brasileira.

Métodos Neste estudo observacional não intervencionista, utilizamos dados retrospectivos da implementação de um algoritmo de tratamento para *P vivax* em 43 unidades de saúde em Manaus e Porto Velho, Brasil. O algoritmo de tratamento consistiu em cloroquina (25 mg/kg durante 3 dias e teste quantitativo de glicose-6-fosfato desidrogenase (G6PD) no local de atendimento seguido de dose única de tafenoquina 300 mg (G6PD normal, idade mínima de 16 anos, não grávida e não amamentando), primaquina 7 dias 0·5 mg/kg por dia (G6PD intermediário ou normal, com pelo menos 6 meses de idade, não grávida e não amamentando ou amamentando há mais de 1 mês), ou primaquina 0·75 mg/kg por semana durante 8 semanas (deficiente de G6PD, idade mínima de 6 meses, não grávida e não amamentando ou amamentando por mais de 1 mês).As recorrências de *P vivax* foram identificadas a partir de ligação probabilística de registros de pacientes de rotina do epidemiológico da malária brasileira sistema de vigilância. A eficácia livre de recorrência no dia 90 e no dia 180 foi estimada usando análise de Kaplan–Meier e taxas de risco (HRs) por análise multivariada. Este ensaio clínico está registrado em ClinicalTrials.gov, NCT05096702, e foi concluído.

Achados Os registros de resultados de 9 de setembro de 2021 a 31 de agosto de 2022 incluíram 5554 pacientes com malária por *P vivax*. Em todos os pacientes tratados de qualquer idade e qualquer status de G6PD, a eficácia livre de recorrência no dia 180 foi de 75·8% (IC 95% 74·0–77·6) com tafenoquina, 73·4% (71·9–75·0) com primaquina de 7 dias e 82·1% (77·7–86·8) com primaquina semanal. Em pacientes normais com G6PD com pelo menos 16 anos de idade, a eficácia livre de recorrência no dia 90 foi de 88·6% (IC 95% 87·2–89·9) naqueles que foram tratados com tafenoquina (n=2134) e 83·5 % (79·8–87·4) naqueles tratados com primaquina por 7 dias (n=370); após ajuste para fatores de confusão, o HR para recorrência após tafenoquina versus primaquina de 7 dias foi de

0·65 (IC 95% 0·49–0·86; p=0·0031), com resultados semelhantes entre os dois tratamentos no dia 180 (log-rank p=0·82). Ao longo de 180 dias, o tempo médio de recorrência em pacientes normais com G6PD com pelo menos 16 anos foi de 92 dias (IQR 76-120) naqueles tratados com tafenoquina e 68 dias (52-94) naqueles tratados com primaquina de 7 dias.

Interpretação Neste cenário do mundo real, a tafenoquina em dose única foi mais eficaz na prevenção da recorrência do *P vivax* em pacientes normais com G6PD com pelo menos 16 anos de idade em comparação com a primaquina de 7 dias no dia 90, enquanto a eficácia global aos 180 dias foi semelhante. Os benefícios para a saúde pública do algoritmo de tratamento de cura radical do *P vivax*, incorporando testes quantitativos de G6PD e tafenoquina, apoiam sua implementação no Brasil e potencialmente em toda a América do Sul.

Financiamento Ministério da Saúde, Secretarias Municipais e Estaduais de Saúde, Fiocruz, Medicines for Malaria Venture, Bill & Melinda Gates Foundation, Newcrest Mining e Governo do Reino Unido.
