# Supplementary material for: Operational effectiveness of tafenoquine and primaquine for the prevention of Plasmodium vivax recurrence in Brazil: a retrospective observational study
Source: Lancet Infect Dis. 2024 Jun;24(6):629–38. doi: 10.1016/S1473-3099(24)00074-4 (PMC7615970; doi:10.1016/S1473-3099(24)00074-4)
Supplement: Supplementary file 2 [file mmc2.pdf]

THE LANCET
Infectious Diseases

Supplementary appendix 2

This appendix formed part of the original submission and has been peer reviewed. We post it as supplied by the authors.

Supplement to: Brito M, Rufatto R, Brito-Sousa JD, et al. Operational effectiveness of tafenoquine and primaquine for the prevention of *Plasmodium vivax* recurrence in Brazil: a retrospective observational study. *Lancet Infect Dis* 2024; published online March 4. https://doi.org/10.1016/S1473-3099(24)00074-4.

Supplementary materials

Operational effectiveness of tafenoquine and primaquine for the prevention of *Plasmodium vivax* recurrence in Brazil: a retrospective observational study

Marcelo Brito, et al.

Table S1 Socio-demographic and baseline characteristics for all treated patients...2
Table S2 Socio-demographic and baseline characteristics by municipality (analysis population)..........................3
Table S3. Median time to recurrence by municipality (analysis population). 5

Table S1 Socio-demographic and baseline characteristics for all treated patients.

Characteristic	Tafenoquine, single dose (N = 2152)	Primaquine, 7 day (N = 3128)	Primaquine, weekly (N = 274)	*P* value[a]
Mean age (SD), years	39·4 (14·1)	34·2 (17·5)	34·7 (16·8)	<0·0001
< 16 years, n (%)	6 (0·3)	560 (17·9)	37 (13·5)	<0·0001
≥ 16 years, n (%)	2146 (99·7)	2568 (82·1)	237 (86·5)	
Median age (IQR), years	39·0 (28·0, 49·0)	33·0 (21·0, 47·0)	33·0 (23·0, 46·0)	<0·0001
G6PD classification, n (%)				<0·0001
Deficient	4 (0·2)	74 (3·8)	249 (92·2)	
Intermediate	8 (0·4)	1207 (61·6)	8 (3·0)	
Normal	2140 (99·4)	680 (34·7)	13 (4·8)	
Missing[b]	0	1167	4	
Mean G6PD activity (SD), U/g Hb	7·6 (1·4)	6·0 (1·6)	3·0 (1·5)	<0·0001
Median G6PD activity (IQR), U/g Hb	7·2 (6·6, 8·1)	5·8 (5·2, 6·7)	2·8 (2·1, 3·6)	<0·0001
Sex, n (%)				0·94
Female	745 (34·6)	1083 (34·6)	92 (33·6)	
Male	1407 (65·4)	2045 (65·4)	182 (66·4)	
Mean weight (SD), kg	75·3 (26·2)	67·1 (28·2)	68·7 (20·7)	<0·0001
Median weight (IQR), kg	72·0 (63·0, 84·0)	68·0 (57·0, 80·0)	70·0 (59·0, 81·2)	<0·0001
Missing weight, n	306	542	30	
Race, n (%)				0·35
Mixed	1844 (85·7)	2683 (85·8)	237 (86·5)	
White	194 (9·0)	317 (10·1)	25 (9·1)	
Black	73 (3·4)	93 (3·0)	7 (2·6)	
Indigenous	25 (1·2)	24 (0·8)	3 (1·1)	
Asian	16 (0·7)	11 (0·4)	2 (0·7)	
Municipality, n (%)				<0·0001
Porto Velho	1359 (63·2)	2422 (77·4)	178 (65·0)	
Manaus	793 (36·8)	706 (22·6)	96 (35·0)	
Facility level, n (%)				<0·0001
High complexity	438 (20·4)	138 (4·4)	23 (8·4)	
Medium complexity	1390 (64·6)	2363 (75·5)	129 (47·1)	
Low complexity	324 (15·1)	627 (20·0)	122 (44·5)	
Education, n (%)				<0·0001
Illiterate	36 (1·7)	59 (1·9)	7 (2·6)	
1st to 5th incomplete	285 (13·3)	474 (15·3)	48 (17·7)	
4th elementary school completed	82 (3·8)	157 (5·1)	15 (5·5)	
5th to 9th elementary school incomplete	317 (14·8)	598 (19·3)	55 (20·3)	
Elementary school completed	326 (15·2)	229 (7·4)	31 (11·4)	
Incomplete high school	354 (16·6)	452 (14·6)	37 (13·7)	
Completed high school	583 (27·3)	799 (25·8)	61 (22·5)	
Incomplete college	46 (2·2)	65 (2·1)	2 (0·7)	
Completed college education	109 (5·1)	149 (4·8)	6 (2·2)	
Not applicable	0 (0·0)	112 (3·6)	9 (3·3)	
Missing[b]	14	34	3	
Occupation in last 15 days, n (%)				<0·0001
Agriculture	243 (8·0)	28 (10·7)	135 (6·5)	
Cattle	8 (0·3)	3 (1·1)	3 (0·1)	
At home	710 (23·2)	66 (25·3)	350 (16·9)	
Tourism	212 (6·9)	8 (3·1)	92 (4·4)	
Gold mining	19 (0·6)	3 (1·1)	32 (1·5)	
Plant exploration	47 (1·5)	7 (2·7)	33 (1·6)	
Hunting/fishing	41 (1·3)	5 (1·9)	14 (0·7)	
Construction roads/dams	3 (0·1)	1 (0·4)	3 (0·1)	
Mining	7 (0·2)	0	4 (0·2)	
Traveler	40 (1·3)	2 (0·8)	37 (1·8)	
Other	1725 (56·5)	138 (52·9)	1368 (66·1)	
Missing[b]	73	13	81	

SD, standard deviation; IQR, interquartile range.
[a] Kruskal-Wallis rank sum test; Pearson's Chi squared test
[b] Missing values were not included in the denominator for percentage calculations.

Table S2 Socio-demographic and baseline characteristics by municipality (analysis population).

Characteristic	Analysis population		*P* value[a]	All patients		*P* value[a]
	**Manaus (N = 846)**	**Porto Velho (N = 1658)**		**Manaus (N =1595)**	**Porto Velho (N = 3959)**	
Mean age (SD), years	38·0 (13·5)	40·0 (14·7)	0·0027	35·1 (16·0)	36·7 (16·6)	0·0024
< 16 years, n (%)	0	0		172 (10·8)	431 (11)	0·9112
≥ 16 years, n (%)	846 (100)	1658 (100)		1423 (89·2)	3528 (89)	
Median age (IQR), years	37·0 (27·0, 47·0)	39.0 (28.2, 51.0)	0·0027	34·0 (24·0, 46·)	36·0 (24·0, 49·0)	0·0024
G6PD classification, n (%)			NA			0·0737
Deficient	0	0		123 (8·6)	204 (6·9)	
Intermediate	0	0		379 (26·5)	844 (28·6)	
Normal	846 (100)	1658 (100)		928 (65·0)	1905 (64·5)	
Missing[b]	0	0		165	1006	
Mean G6PD activity (SD), U/g Hb	7·8 (1·5)	7·5 (1·4)	<0·0001	6·7 (2·0)	6·6 (1·8)	0·0134
Median G6PD activity (IQR), U/g Hb	7·5 (6·8, 8·3)	7·1 (6·6, 8·0)	<0·0001	6·7 (5·6, 7·8)	6·5 (5·6, 7·5)	0·0134
Sex, n (%)			0·28			0·1102
Female	316 (37·4)	583 (35·0)		577 (36·2)	1343 (33·9)	
Male	530 (62·6)	1075 (65·0)		1018 (63·8)	2616 (66·1)	
Mean weight (SD), kg	73·5 (18·9)	75·4 (28·2)	0·0063			0·0001
Median weight (IQR), kg	71·0 (62·0, 82·2)	72·0 (63·0, 85·0)	0·0063	68·0 (59·0, 80·0)	70·0 (60·0, 82·0)	0·0001
Missing weight, n	58	311		89	789	
Race, n (%)			<0·0001			<0·0001
Mixed	780 (92·2)	1370 (83·0)		1465 (91·8)	3299 (83·3)	
White	22 (2·6)	205 (12·0)		48 (3·0)	488 (12·3)	
Black	21 (2·5)	61 (3·7)		41 (2·6)	132 (3·3)	
Indigenous	11 (1·3)	17 (1·0)		21 (1·3)	31 (0·8)	
Asian	12 (1·4)	5 (0·3)		20 (1·3)	9 (0·2)	
Facility level, n (%)			<0·0001			<0·0001
High complexity	457 (54·0)	0 (0)		599 (37·6)	0 (0)	
Medium complexity	276 (32·6)	1393 (84·0)		354 (22·2)	719 (18·2)	
Low complexity	113 (13·4)	265 (16·0)		642 (40·3)	3240 (81·8)	
Education, n (%)			<0·0001			<0·0001
Illiterate	5 (0·6)	36 (2·2)		10 (0·6)	92 (2·4)	
1st to 5th incomplete	53 (6·3)	263 (16·0)		155 (9·7)	652 (17)	
4th elementary school completed	27 (3·2)	68 (4·2)		69 (4·3)	185 (4·7)	
5th to 9th elementary school incomplete	82 (9·7)	303 (19·0)		195 (12·2)	775 (19·8)	
Elementary school completed	242 (28·7)	111 (6·8)		347 (21·8)	239 (6·1)	
Incomplete high school	183 (21·7)	231 (14·0)		304 (19·1)	539 (13·8)	
Completed high school	219 (25·9)	475 (29·0)		398 (25·0)	1045 (26·7)	
Incomplete college	19 (2·3)	33 (2·0)		36 (2·3)	77 (2·0)	
Completed college education	14 (1·7)	114 (7·0)		27 (1·7)	237 (6·1)	
Not applicable	0	0		52 (3·3)	69 (1·8)	
Missing[b]	2	24		2	49	
Occupation in last 15 days, n (%)			< 0.0001			<0·0001
Agriculture	41 (5·2)	128 (7·9)		111 (7·3)	295 (7·6)	
Cattle	0	4 (0·2)		5 (0·3)	9 (0·2)	
At home	86 (10·9)	377 (23·0)		209 (13·7)	917 (23·7)	
Tourism	7 (0·9)	123 (7·6)		26 (1·7)	286 (7·4)	
Gold mining	25 (3·2)	10 (0·6)		36 (2·4)	18 (0·5)	
Plant exploration	3 (0·4)	38 (2·3)		6 (0·4)	81 (2·1)	
Hunting/fishing	2 (0·3)	18 (1·1)		6 (0·4)	54 (1·4)	
Construction roads/dams	3 (0·4)	1 (<0·1)		4 (0·3)	3 (<0·1)	
Mining	3 (0·4)	3 (0·2)		4 (0·3)	7 (0·2)	
Traveler	35 (4·4)	11 (0·7)		51 (3·3)	28 (0·7)	
Other	585 (74·1)	912 (56·0)		1066 (69·9)	2165 (56·0)	
Missing[b]	56	33		71	96	

SD, standard deviation; IQR, interquartile range.
[a] Kruskal-Wallis rank sum test; Pearson’s Chi squared test
[b] Missing values were not included in the denominator for percentage calculations.

Table S3. Median time to recurrence by municipality (analysis population).

Municipality	Day 90		Day 180	
	Tafenoquine, single dose	Primaquine, 7 day	Tafenoquine, single dose	Primaquine, 7 day
Manaus	75 (64, 82)	60 (48, 79)	93 (75, 119)	70 (48, 85)
Porto Velho	75 (66, 82)	62 (42, 70)	92 (77, 122)	68 (54, 98)

Values are median (interquartile range).
